# Nabilone as Part of the Holistic Treatment in Early Onset Alzheimer's Dementia: A Case Study

**DOI:** 10.1192/bjo.2024.687

**Published:** 2024-08-01

**Authors:** Charvi Saraswat, Jiedi Lei, Tharun Zacharia, Anne M. Bonnici Mallia

**Affiliations:** 1 South London and Maudsley NHS Foundation Trust, London, United Kingdom; 2 Institute of Psychiatry, Psychology and Neuroscience (IoPPN), King's College London, London, United Kingdom; 3 University of Oxford, Oxford, United Kingdom

## Abstract

**Aims:**

Optimal management of Behavioural and Psychological symptoms of Dementia (BPSD) remains challenging. This report describes using nabilone, a synthetic cannabinoid, in a 61-year-old woman with Alzheimer's dementia (AD) experiencing progressive BPSDs.

**Methods:**

AM was diagnosed with AD in February 2019 and prescribed donepezil and mirtazapine. In August 2021, her behaviour deteriorated, becoming paranoid, repeatedly pacing and developing expressive aphasia. Behaviours further declined leading to an admission to our dementia ward under the Mental Health Act 2007 in January 2022. AM showed limited response to medications including risperidone and mirtazapine which were switched to olanzapine and citalopram. “Controlled falls” were observed, during which AM placed herself suddenly onto the floor. In February 2022, she was witnessed having a self-terminating generalised tonic clonic seizure (GTCS) lasting 3 minutes and later witnessed having three more seizures. Computed tomography excluded acute intracranial pathology. She had no previous history of seizures. An electroencephalogram displayed focal slowing over the frontal region greater on the right and presence of sharp, transient, sharpened slow wave, triphasic waves and reported that epileptiform discharges can be seen in AD in the absence of epilepsy.

Behavioural charts, Cohen Mansfield Agitation Inventory (CMAI) and Neuropsychiatric inventory (NPI) questionnaires were used to monitor response. Decision was made to trial Nabilone in April 2022 due to minimal improvement. Nabilone was started at 0.25 mg daily and up-titrated by 0.25 mg fortnightly based on the response. Over the subsequent month there was a measurable improvement. This was temporarily halted due to issues with nabilone supply, together with cessation of lorazepam, showing worsening in behaviours. Nabilone was eventually restarted and increased to 1 mg once daily with promising effect.

**Results:**

There was a notable qualitative improvement in AM's engagement and communication with family and staff. Prior to treatment the frequency of aggressive incidents ranged from 25–35, reducing to five to ten incidents per day. Controlled falls largely ceased. The NPI Caregiver distress score dropped from 21 to 8 over three months; Frequency and severity scores dropped from 73 to 40 during the same period. CMAI scores dropped from 86 to 64 over two months.

**Conclusion:**

We describe a measurable improvement in BPSDs and quality of life in a patient with severe AD. Reduction in irritability, agitation and improvements in sleep were observed after initiating nabilone. The mechanism of nabilone via CB1 agonism has shown to be neuroprotective and anti-inflammatory. This indicates a promising treatment for BPSDs.

